# Sulphuric Acid by the Mouth in Furunculosis

**Published:** 1909-02-06

**Authors:** 


### SULPHURIC ACID BY THE MOUTH IN FURUNCULOSIS.

The influence of the general health, and particularly of the state of the digestion, upon the occurrence of boils and carbuncles is well enough known. There are of course special causes of furunculosis, and of severe carbuncles, diabetes mellitus, for instance, for which very special treatment is required; but besides these special causes there are a great number of cases in which indigestion, or the fact of being "run down," lead to an outcrop of the most troublesome and recurrent boils upon the neck, back, face, and elsewhere.

There are, as is well known, many different forms of local and general treatment that may be employed, from simply squeezing out the contents of each furuncle as it arises, to hypodermic injection of a vaccine prepared from the staphylococci cultivated from the patient's own lesions. There is one line of treatment, however, which is sometimes neglected, although it is one of extreme simplicity, and this is by the administration orally of that fine stomachic—dilute sulphuric acid B.P. It may or may not be combined with liquid extract of nux vomica, with quinine, or other tonic, but this is by no means necessary.

The main point in this method is to give sufficient of the dilute sulphuric acid and to give it often enough. Twenty to thirty minim doses, well diluted with water, repeated four hourly, should be ordered at once, and the prescription persisted with for at least ten days or more after the boils are well on the way to complete disappearance. Local treatment of the latter need not be drastic, but if a series of cases of acute affection by boils or carbuncles be treated with these large and frequent doses of dilute sulphuric acid, and compared with another series treated in other ways, no doubt will remain as to the efficacy of the dilute sulphuric acid treatment.

If it is desired to avoid the idea of constant medication, and yet to continue the administration of sulphuric acid, the latter may be made up into an artificial lemonade in a way similar to that employed to prevent plumbism in the Potteries, and amongst workers in lead.

